# Mediastinal Lymphoma in 70 Dogs Treated With Lomustine or Anthracycline‐Based Multi‐Agent Chemotherapy

**DOI:** 10.1111/vco.70062

**Published:** 2026-03-31

**Authors:** Diogo Machado, Jose Alvarez Picornell, Isabel Amores‐Fuster, Sarah Benjamin, Thomas Kearns, Alenka Lavra Zajc, Yike Bing, Katie A. McNaught, Maria Sava, Charlotte Johnston, Beth Rolf, Andrew D. Yale

**Affiliations:** ^1^ Department of Clinical Science and Services Royal Veterinary College Hatfield UK; ^2^ Department of Small Animal Clinical Sciences University of Liverpool Neston UK; ^3^ Davies Veterinary Specialists Hitchin UK; ^4^ Department of Veterinary Medicine, Queen's Veterinary School Hospital, School of Biological Sciences University of Cambridge Cambridge UK; ^5^ Northwest Veterinary Specialists Runcorn UK; ^6^ AURA Veterinary Guildford UK; ^7^ School of Veterinary Medicine, Small Animal Hospital, College of Medical, Veterinary, and Life Sciences University of Glasgow Glasgow UK; ^8^ North Downs Specialist Referrals Bletchingley UK; ^9^ Southern Counties Veterinary Specialists Ringwood UK; ^10^ Bristol Vet Specialists Bristol UK

**Keywords:** canine, chemotherapeutics, haematopoietic, oncology, T‐cell

## Abstract

Primary mediastinal lymphoma is rare in dogs and literature exploring this disease is limited. Therefore, the aim of this study was to describe presentation, treatment and outcome in a large cohort of dogs with mediastinal lymphoma and explore prognostic factors including chemotherapy protocol. This retrospective multi‐institute study included 70 dogs with primary mediastinal lymphoma treated with lomustine‐based (LOP/LOPP) or anthracycline‐based (CHOP/CEOP) chemotherapy. Most immunophenotyped cases were of T‐cell lineage (95.6%). The majority were substage *b* (90%) and hypercalcaemia was noted in 69.1% of dogs. Clinical and objective response rates to chemotherapy were 92.7% and 97.9%, respectively, with 76.6% of dogs achieving a complete response. Median progression free survival (PFS) was 132 days (95% CI 83–181), and median overall survival time (OST) was 223 days (95% CI 175–271). The 6‐month, 1‐year, and 2‐year survival rates were 55.7%, 22.9%, and 15.7%, respectively. On multivariable analysis, factors associated with longer PFS included hypercalcaemia (*p* = 0.041), chemotherapy‐induced neutropenia (*p* = 0.014) and CD4+/CD8− immunophenotype (*p* = 0.004). Neutropenia at diagnosis was associated with shorter PFS (*p* = 0.015) and OST (*p* = 0.004). Other factors associated with shorter OST included granular morphology (*p* = 0.023) and CD4+/CD8+ immunophenotype (*p* = 0.004). Chemotherapy‐induced neutropenia was associated with improved OST (*p* = 0.042). Differences in outcome between anthracycline‐ or lomustine‐based chemotherapy protocols were not statistically significant. Overall, the prognosis for primary mediastinal lymphoma in dogs is poor to fair when treated with multi‐agent chemotherapy. This is the second study associating hypercalcaemia with improved PFS in dogs with non‐indolent T‐cell lymphoma. Results also suggest prognostic significance of specific CD4/CD8 expression patterns.

## 1 Introduction

Lymphoma is the most common haematopoietic malignancy in dogs [1, 2]. Cranial mediastinal involvement is sometimes encountered as part of multicentric disease (35.9%–54.1%), particularly with non‐indolent T‐cell lymphomas [3, 4, 5, 6]. Primary mediastinal origin is also recognised as a rare but distinct anatomical form, almost exclusively of T‐cell lineage [7].

In humans, mediastinal lymphoma occurs in two main forms: primary mediastinal large B‐cell lymphoma and T‐lymphoblastic lymphoma (T‐LBL). Mediastinal T‐LBL predominantly affects young males, commonly presenting with a mediastinal mass and supra‐diaphragmatic lymphadenopathy [8, 9, 10, 11]. In dogs, an association between the lymphoblastic World Health Organisation (WHO) subtype and mediastinal lymphoma has also been suggested [12, 13].

The CHOP chemotherapy protocol has been preferred for most high‐grade lymphomas in dogs, generally with shorter duration of response and survival times in T‐cell compared to B‐cell lymphoma. However, recent studies have suggested that protocols incorporating lomustine may offer similar or superior outcomes in T‐cell lymphoma [6, 14, 15, 16, 17, 18, 19].

Moore et al. described a population of dogs with primary mediastinal lymphoma, including treatment in 36 cases, and reported a median PFS of 144 days and OST of 194 days for CHOP‐treated dogs. The CHOP protocol was associated with improved outcomes but lomustine was not included in any of the other chemotherapy protocols [7]. There is mounting evidence to suggest that flow cytometry expression patterns carries prognostic significance in canine T‐cell lymphoma, which was not investigated in this previous study [6, 20, 21]. This might be of particular interest for lymphoid malignancies arising in the thymic region, which might express characteristic immunophenotypes [8, 12, 13, 22, 23].

The main aim of this study was to describe clinical presentation, immunophenotypical characteristics, response to treatment, and outcome in the largest cohort of dogs with mediastinal lymphoma to date, treated with multi‐agent chemotherapy. Secondary aims were to identify potential prognostic factors and compare the efficacy of anthracycline‐based versus lomustine‐based chemotherapy protocols.

## 2 Materials and Methods

### 2.1 Study Design and Case Selection

Medical records from 10 institutions were retrospectively reviewed (2009–2023). Dogs were eligible for inclusion if they had a mediastinal mass at diagnosis and primary disease burden within the thoracic cavity, a cytologic or histologic diagnosis of intermediate or large cell lymphoma from the mediastinal mass or pleural effusion, and no extra‐nodal tissue involvement (except liver, spleen, thymus, or bone marrow). All patients were required to have received first‐line treatment with one of the following chemotherapy protocols: CHOP (cyclophosphamide, doxorubicin, vincristine, prednisone), CEOP (cyclophosphamide, epirubicin, vincristine, prednisone), LOP (lomustine, vincristine, prednisone), or LOPP (lomustine, vincristine, procarbazine, prednisone) with or without L‐asparaginase.

Dogs were excluded if the mediastinal mass was accompanied by marked peripheral lymphadenopathy cranial to the diaphragm or peripheral lymphadenopathy in other locations. Confirmed or suspected involvement of peripheral or intrathoracic lymph nodes cranial to the diaphragm based on cytology, histopathology or presence of lymphadenopathy was permitted, as is documented in people with anterior mediastinal lymphoma; intra‐abdominal lymphadenopathy was also permitted [24]. Pre‐treatment with corticosteroids for a maximum of 7 days prior to chemotherapy was permitted. Ethical approval was obtained from the Royal Veterinary College's Clinical Research Ethical Review Board (URN SR2022–0172).

### 2.2 Data Collection

Patient data collected included demographic information (age, breed, sex, neuter status, body weight), presenting clinical signs, physical examination findings, relevant comorbidities, steroid pre‐treatment and method of diagnosis (cytology and/or histopathology). Clinicopathologic data collected included immunophenotyping methods (flow cytometry, immunohistochemistry, immunocytochemistry, clonality testing), haematology, serum biochemistry, bone marrow aspirates/biopsies, liver/spleen cytology, and diagnostic imaging findings. Differentiation between B‐cell or T‐cell phenotype on flow cytometry was based on patterns of marker expression including known possible aberrant expressions. Given the constraints of the inclusion criteria, WHO clinical stages were not assigned. All cases were categorised by WHO substage (*a* or *b*) [25].

Haematological and serum biochemical abnormalities were defined according to each institution's reference intervals. Clinical pathology reports of blood smear evaluations were reviewed for the presence of circulating neoplastic cells. Neoplastic cell morphology characteristics including size, determined either by flow cytometry or nuclear size (intermediate = 1.5–2× red blood cell [RBC] diameter, large = > 2× RBC diameter), lymphoblastic features, large granular lymphocyte (LGL) morphology, and histopathological grade were documented when available. Low‐, medium‐, or high‐grade were defined as 0–5, 6–10 and > 10 mitoses, respectively, per 400× magnification field or 2.37 mm2 on histopathology samples as previously described [26]. Lymphoblastic morphology was defined as intermediate‐sized cells with round/convoluted nuclei, finely ‘stippled’, ‘granular’ or ‘dusty’ chromatin, inconspicuous nucleoli and small ring of cytoplasm; LGL was defined by the presence of azurophilic cytoplasmic granules [12, 13, 27].

Information recorded regarding the first‐line chemotherapy protocol included chemotherapy drugs, intended dosages, administration schedule, and adverse events graded according to the Veterinary Cooperative Oncology Group Common Terminology Criteria for Adverse Events (VCOG‐CTCAE v2) [28]. Rescue and maintenance protocols were also recorded.

Outcome data recorded included response to first‐line protocols and date of progression, death, or last follow‐up. Response was assessed using the VCOG canine response evaluation criteria for solid tumours in dogs (RECIST v1.0) as determined by repeated imaging and cytology results (when deemed appropriate) [29]. The objective response rate (ORR) was defined as the percentage of dogs that achieved either a complete (CR) or partial (PR) response. Clinical response was defined as CR, PR and/or improvement in clinical signs.

### 2.3 Data Analysis

Categorical variables were presented as frequency and proportion. Continuous data normality was assessed by the Shapiro–Wilk test. Normally distributed data were reported as mean and standard deviation and non‐normal data as median and range, and/or 95% confidence intervals (CI).

At the time of data collection, each patient was recorded as alive, dead, or lost to follow‐up (LTFU). Progression‐free survival (PFS) was defined as the time between chemotherapy initiation to disease progression or death. Overall survival time (OST) was defined as the time between chemotherapy initiation to death. Dogs LTFU or alive without documented progression at the time of data abstraction were censored from PFS analysis, whilst dogs alive or LTFU were censored from OST analysis. Kaplan–Meier survival analysis was used to estimate median PFS and OST. Patients were classed as long‐term survivors if alive ≥ 450 days after the start of chemotherapy, corresponding to the plateau of the right tail of the Kaplan–Meier survival curve and representing the top quintile (approximately 20%) of survival times.

Univariable and multivariable backward stepwise Cox regression analysis was used to test variables for an association with progression and survival. Variables with *p* ≤ 0.1 in univariable analysis were included in the multivariable models. Binary logistic regression was used to investigate variables associated with clinical response, complete response, and long‐term survival. Results were presented as odds ratios (OR) or hazard ratios (HR) with 95% CI. A *p*‐value ≤ 0.05 was considered statistically significant.

Variables assessed included: age, breed (boxer, Labrador retriever or purebreed vs. others), weight, WHO substage, individual haematologic and biochemical abnormalities, presence of pleural effusion, presence of circulating neoplastic cells, individual cytologic features (cell size, LGL or lymphoblastic cytomorphology), histological grade, administration of L‐asparaginase, treatment with steroids prior to chemotherapy, first‐line chemotherapy protocol (CHOP/CEOP, LOP or LOPP), treatment with rescue chemotherapy following progression, resolution of creatinine elevation, occurrence of chemotherapy‐induced neutropenia, and specific expression patterns on flow cytometry, including MHC‐II expression for all cases and individual expression of CD79a, CD3 or CD5 as well as specific expression patterns for CD4 and CD8 (CD4+/CD8−, CD4−/CD8+, CD4+/CD8+, CD4−/CD8−) in lymphomas of T‐cell lineage. Patients who received only the first chemotherapy treatment in a protocol were excluded from protocol comparisons. For statistical purposes, ‘hypercalcaemia’ was defined as either total or ionised hypercalcaemia.

All statistical analyses were performed using IBM SPSS Statistics version 30.0 (IBM Corp., Armonk, NY, USA).

## 3 Results

### 3.1 Patient Characteristics

**FIGURE 1:**
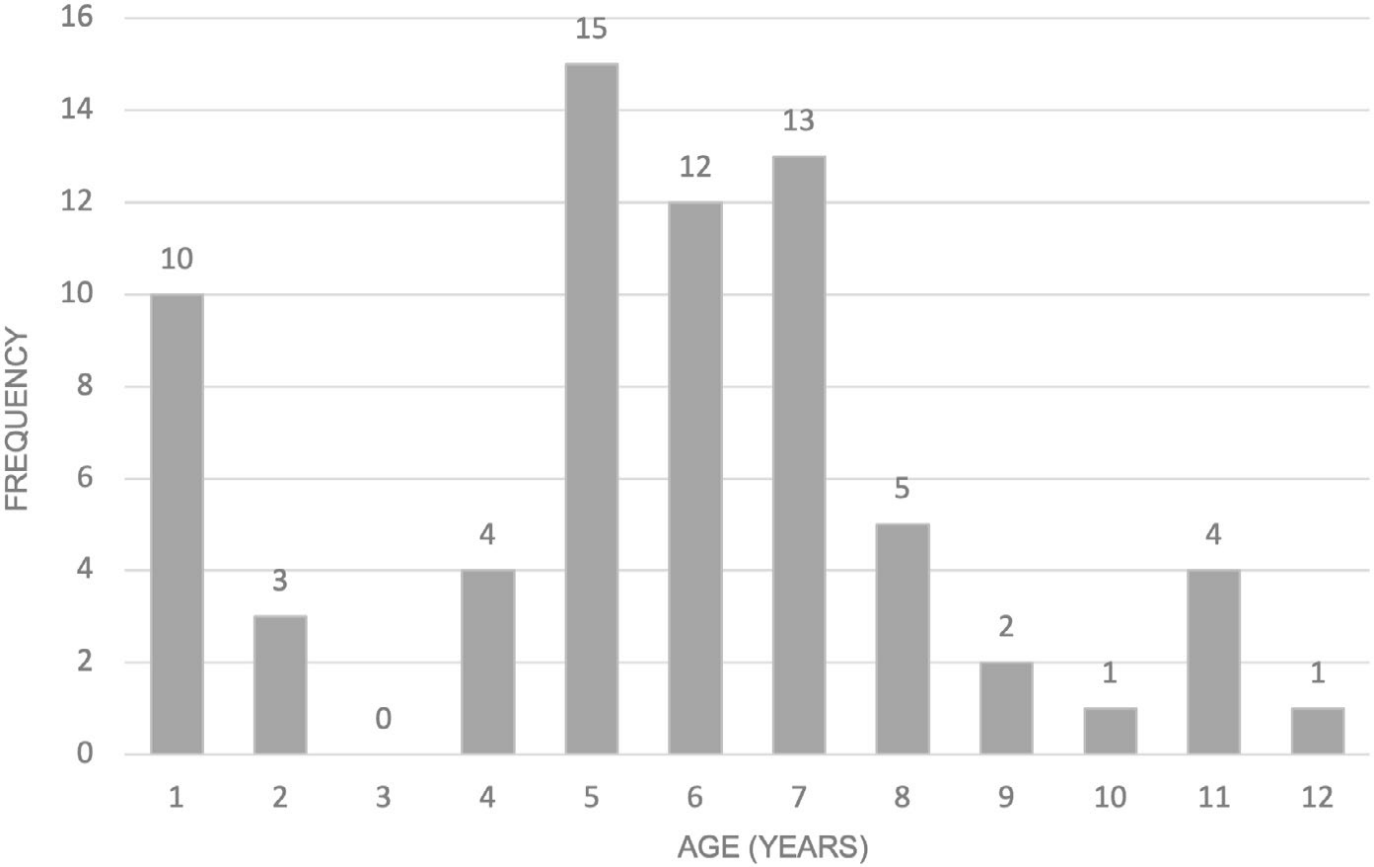
Age of dogs diagnosed with mediastinal lymphoma. A bimodal distribution is evident, with peaks at approximately 1 and 5–7 years of age.

A total of 70 dogs met the inclusion criteria. Median body weight was 28 kg (range, 4–65.5) and the median age was 6 years (range, 0.8–12) with a bimodal distribution illustrated in Figure 1. There were 14 entire males (20%), 37 neutered males (52.9%), four entire females (5.7%), and 15 neutered females (21.4%). Twenty‐five breeds were represented, with the most common being Labrador retriever (*n* = 17 [24.3%]), boxer (*n* = 11 [15.7%]), and crossbreed (*n* = 14 [20%]).

Sixty‐three dogs (90%) were classified as substage *b*. Median duration of clinical signs prior to diagnosis was 14 days (range, 1–60), most commonly lethargy (*n* = 44 [62.9%]), inappetence (*n* = 39 [55.7%]), respiratory signs including cough, tachypnoea or dyspnoea (*n* = 32 [45.7%]), polyuria/polydipsia (*n* = 36 [51.4%]), vomiting (*n* = 21 [30%]) and regurgitation (*n* = 7 [10%]). On examination, signs of cranial vena cava syndrome were evident in seven dogs (10%). Mandibular, superficial cervical or axillary lymphadenopathy was reported in 12 dogs (17.1%); lymphoma was confirmed in two of three that had cytology.

### 3.2 Clinicopathologic and Staging Findings

Haematology and serum biochemistry panels were available in 66 and 62 dogs, respectively. Haematological abnormalities included monocytosis (28.8%), neutrophilia (18.2%), thrombocytopenia (13.6%), anaemia (9.0%), neutropenia (4.6%), eosinophilia (4.6%), and lymphocytosis (1.5%). Serum biochemistry abnormalities included increased ALT (29.0%), increased creatinine (25.8%), hypoalbuminaemia (3.2%), and hyperglobulinaemia (3.2%). Total calcium was increased in 37 dogs (58.7%) and ionised calcium, measured in 57 dogs, was increased in 43 (75.4%). C‐reactive protein was elevated in 42.9% (*n* = 7). On blood smear evaluation (*n* = 55) circulating neoplastic lymphocytes were noted in two cases (3.6%) and suspected in one (1.8%).

All dogs underwent thoracic imaging including radiographs (*n* = 39 [55.7%]), computed tomography (CT) (*n* = 21 [30%]), both thoracic radiographs and CT (*n* = 6 [8.6%]) or ultrasound (*n* = 4 [5.7%]). The widest measurement for the mediastinal mass was known from CT or thoracic radiographs for 29 dogs, with a median of 8.14 cm (range, 2.6–23.0). Pleural effusion was present in 32 dogs (45.7%), with three (4.3%) also having mild pericardial effusion. Intrathoracic lymphadenopathy reported for 18 dogs (25.7%) included sternal (*n* = 7 [38.9%]), tracheobronchial (*n* = 4 [22.2%]), multiple (*n* = 4 [22.2%]) or unspecified (*n* = 3 [16.7%]) lymph nodes.

Abdominal imaging, performed in 53 dogs (75.7%), included ultrasound (*n* = 27 [38.6%]), CT (*n* = 24 [34.3%]) or radiographs (*n* = 9 [12.9%]). Intra‐abdominal lymphadenopathy was reported in 20 cases (37.7%). The liver and spleen were sampled in 17 cases, whilst liver or spleen alone were sampled in five and seven cases, respectively. Lymphoma infiltration was confirmed or suspected in six of 22 livers (27.3%) and nine of 24 spleens sampled (37.5%). A bone marrow aspirate was performed in one dog and was unremarkable.

### 3.3 Cytologic, Histological, and Immunophenotypic Characteristics

Cytology of the mediastinal mass was performed in 69 cases (98.6%), with one dog (1.4%) diagnosed through pleural effusion analysis. Results were compatible with lymphoma in 59 cases (84.3%), suspicious in nine (12.9%), inconclusive in one (1.4%), and initially suggestive of thymoma in another case. Tru‐cut biopsies were performed in 16 patients (22.9%), with histopathology confirming the diagnosis in 15 cases and immunohistochemistry being required for final diagnosis in one. Grade was available in 12 cases, including nine (75%) low‐grade, one (8.3%) intermediate‐grade, and two (16.7%) high‐grade tumours. Cell size was reported in 58 cases and was categorised as intermediate in 17 (29.3%), large in 13 (22.4%), and intermediate‐to‐large in 28 (48.3%). Granular morphology was noted in six dogs (8.6%). Out of 45 cases with sufficient morphological information, 10 (22.2%) were consistent with lymphoblastic morphology.

Immunophenotype was investigated in 49 cases (70.0%) using flow cytometry (*n* = 24), immunohistochemistry (*n* = 11), immunocytochemistry (*n* = 4) and/or PCR for antigen receptor rearrangement (PARR, *n* = 14). Of the 45 cases with conclusive immunophenotype, 43 (95.6%) were T‐cell and two (4.4%) were B‐cell lymphomas. Table 1 summarises the flow cytometry results for 21 patients with available reports.

**TABLE 1:** Flow cytometry results from 21 dogs with mediastinal lymphoma.

Case	Sample	Cell lineage	Phenotype
1	Spleen	T	CD18+, CD5+, CD3+, CD4+, MHC‐II+; CD8−, CD21−
2	Mediastinal mass	T	CD18+, CD45+, CD5+; CD3−, CD4−, CD8−, CD21−
3	Mediastinal mass	T	CD18+, CD45+, CD3+, CD4+, MHC‐II+; CD8−
4	Mediastinal mass	T	CD18+, CD45+, CD5+, CD3+, CD4+; MHC‐II−
5	Mediastinal mass	T	CD5+, CD4+; MHC‐II+
6	Mediastinal mass	T	CD18+, CD45+, CD5+, CD3+, CD4+, CD8+; CD11d−, CD21−, CD79a−, CD34−, MHC‐II−
7	Mediastinal mass	T	CD45+, CD3+, CD4+, CD79a+, MHC‐II+; CD5−, CD8−, CD21−, CD34−
8	Mediastinal mass	T	MHC‐II+; CD5−, CD3−, CD4−, CD8−, CD21−
9	Mediastinal mass	T	CD45+, CD5+, CD3+, CD4+; CD8−, CD21−, CD79a−, CD34−, MHC‐II−
10	Mediastinal mass	T	CD45+, CD5+, CD3+, CD4+, CD79a+; CD8−, CD21−, MHC‐II−, CD34−
11	Mediastinal mass	B	CD5−, CD4−, CD8−, CD21−, CD79a+; CD45−, CD34−, MHC‐II−
12	Mediastinal mass	Inconclusive	CD45+, CD5+, CD4+, CD21+; CD3−, CD8−, CD79a−, CD34−, MHC‐II−
13	Superficial cervical lymph node	T	CD45+, CD3+, CD4+, CD79a +; CD5−, CD8−, CD21−, CD34−, MHC‐II−
14	Pleural effusion	T	CD45+, CD3+; CD5−, CD4−, CD8−, CD21−, CD79a−, CD34−, MHC‐II−
15	Mediastinal mass	B	CD45+, CD79a+; CD5−, CD3−, CD4−, CD8−, CD21−, CD34−, MHC‐II−
16	Mediastinal mass	T	CD45+, CD5+, CD4+, CD8+, CD79a+; CD3−, CD21−, CD34−, MHC‐II−
17	Mediastinal mass	T	CD45+, CD5+, CD3+; CD4−, CD8−, CD21−, CD79a−, CD34−, MHC‐II−
18	Mediastinal mass	T	CD18+, CD45+, CD5+, CD3+, CD4+, MHC−II+; CD11d−, CD8−, CD21−, CD79a−, CD34−
19	Pleural effusion	T	CD45+, CD5+, CD3+, CD4+, CD8+; CD21−, CD79−, CD34−, MHC‐II−
20	Superficial cervical lymph node	T	CD45+, CD5+; CD4−, CD8−, CD21−, CD79b−, CD34−
21	Pleural effusion	T	CD45+, CD5+, CD3+; CD4−, CD8−, CD21−, CD79a−, CD34−, MHC‐II−

*Note*: Patient ‘8’ revealed T‐cell receptor rearrangement on PARR; no other patients with flow cytometry available had additional immunophenotyping tests performed.

### 3.4 Treatment and Response

Sixty‐five dogs (92.9%) were treated with one of four chemotherapy protocols: CHOP (*n* = 15 [21.4%]), CEOP (*n* = 15 [21.4%]), LOP, (*n* = 23 [32.9%]) or LOPP (*n* = 12 [17.1%]). The remaining five dogs (7.1%) received only one initial vincristine dose due to subsequent disease progression or owner decision.

Anthracycline‐based chemotherapy consisted of intravenous (IV) vincristine on days one and 15, IV or oral (PO) cyclophosphamide on day eight, and doxorubicin or epirubicin on day 22. Dosages and modifications were implemented at the discretion of the supervising clinician. A total of four cycles were planned, and the intended protocol duration was 25 weeks in 26 dogs (86.7%) and 19 in four (13.3%). LOP and LOPP protocols varied. Eight patients (22.9%) received weekly vincristine with lomustine every 3–4 weeks for 20–23 weeks; 27 patients (77.1%) received vincristine (day 1) and lomustine (day 8) in 22‐day cycles for 6–8 cycles (total duration of 18–24 weeks). Procarbazine (LOPP, *n* = 12) was given at a median dose of 45.3 mg/m^2^ (range, 25–52.5) daily for 7–14 days (median, 13), initiated on the first cycle for eight patients (67%) or on the second cycle for four patients (33%). None of the patients with initiation in the second cycle had disease progression documented at this time.

L‐asparaginase was administered in 26 cases (37.1%); nine dogs received it alongside LOP or LOPP and 17 dogs alongside CHOP or CEOP protocols. All dogs received prednisolone, and S‐adenosylmethionine (SAMe) was administered in 24 (68.6%) dogs receiving LOP or LOPP. Maintenance chlorambucil/methotrexate protocols were employed in three patients, following completion of LOPP in two cases and LOP in one case.

A clinical response was observed in 63 of the 68 patients where this information was available (92.7%). This included all dogs receiving CHOP, LOP or LOPP and 13 dogs (86.7%) receiving CEOP. Forty‐eight dogs (68.6%) had data available regarding objective response, for which first diagnostic imaging was performed at a median of 54 days after the start of chemotherapy. The objective response rate was 97.9%, including CR in 37 dogs (77.1%) and PR in 10 (20.8%). Progressive disease was noted in one dog (2.1%). This included 77.8% CR and 22.2% PR in those receiving CHOP, 61.5% CR, 30.8% PR and 7.7% PD with CEOP, 83.3% CR and 16.7% PR with LOP, and 87.5% CR and 12.5% PR with LOPP.

Grade 3–4 toxicities included haematological toxicities in 18 dogs (25.7%), gastrointestinal toxicities in five dogs (7.1%), and ALT elevations in 10 dogs (28.6%) receiving lomustine‐based protocols. Forty‐nine dogs (70%) did not complete the chemotherapy protocol; the key reasons included disease progression (*n* = 28 [57.1%]), adverse events (*n* = 13 [26.5%]), or lack of satisfactory response or clinical improvement (*n* = 2 [4.1%]). Seven of the nine interruptions in lomustine‐based protocols were due to grade 3–4 ALT elevations.

Rescue protocols were initiated at the time of progression in 35 (50%) dogs (median 1; range 1–5 protocols), including dexamethasone/melphalan/actinomycin‐d/cytarabine (*n* = 8), LOP/LOPP (*n* = 6), vinca alkaloid/cyclophosphamide‐based protocols (*n* = 4), CHOP (*n* = 2) and various other multi‐agent protocols (*n* = 11). Single‐agent rescue protocols included l‐asparaginase (*n* = 8), doxorubicin (*n* = 6), lomustine (*n* = 5) and other individual agents (*n* = 7).

### 3.5 Outcome and Prognostic Factors

Median PFS for all dogs was 132 days (95% CI 83–181). When stratified by protocol, median PFS was 173 days (95% CI 91–255) for dogs treated with CHOP, 113 days (95% CI 33–193) for CEOP, 187 days (95% CI 159–215) for LOP and 114 days (95% CI 80–148) for LOPP, with no statistically significant differences between groups (*p* = 0.628). Median OST for all dogs was 223 days (95% CI 175–271), with 6‐month, 1‐year, and 2‐year survival rates of 55.7%, 22.9%, and 15.7%, respectively. Median OST was 223 days (95% CI 154–292) for dogs treated with CHOP, 341 days (95% CI 231–451) for CEOP, 222 days (95% CI 140–304) for LOP, and 128 days (95% CI 55–201) for LOPP. These differences were not statistically significant (*p* = 0.305). Fourteen dogs (20.0%) were classified as long‐term survivors (≥ 450 days). This included none of the dogs receiving a single vincristine treatment, two (13.3%) treated with CHOP, one (6.7%) with CEOP, six (26.1%) with LOP, and five (41.7%) with LOPP. These differences were not statistically significant (*p* = 0.320). Differences in clinical or complete response rates between treatment groups were also not statistically significant (*p* = 0.735 and *p* = 0.887, respectively).

Factors associated with PFS and OST on univariable analysis (*p* ≤ 0.10), and therefore used in multivariable analysis, are displayed in Table 2. Full univariable analysis results are provided in the Supporting Information. On multivariable analysis, factors associated with longer PFS included hypercalcaemia (*p* = 0.041; HR 0.19 [95% CI 0.04–0.94]), chemotherapy‐induced neutropenia (*p* = 0.014; HR 0.11 [95% CI 0.02–0.64]), and a CD4+/CD8− phenotype (*p* = 0.004; HR 0.09 [95% CI 0.02–0.47]) compared to other CD4/CD8 expression patterns in T‐cell lymphomas, all of which were either CD4+/CD8+ or CD4−/CD8−. Neutropenia at diagnosis (*p* = 0.015; HR 16.70 [95% CI 1.73–161.26]) was associated with shorter PFS.

**TABLE 2:** Univariable Cox regression analysis results (*p* ≤ 0.10) included in multivariable analysis for prognostic factors for progression and survival in dogs with mediastinal lymphoma.

Progression free survival				
Parameter (number of dogs)		PFS (days)	*p*	HR (95% CI)
Lymphoblastic morphology	No (*n* = 35)	133	0.038	2.18 (1.05–4.57)
	Yes (*n* = 10)	48		
T‐cell CD4+/CD8− phenotype	No (*n* = 9)	70	0.031	0.29 (0.10–0.90)
	Yes (*n* = 7)	345		
Neutropenia at diagnosis	No (*n* = 63)	132	0.029	3.80 (1.15–12.62)
	Yes (*n* = 3)	48		
Hypercalcaemia	No (*n* = 20)	70	0.016	0.51 (0.29–0.88)
	Yes (*n* = 48)	173		
Chemotherapy‐induced neutropenia	No (*n* = 24)	90	0.050	0.58 (0.34–1.00)
	Yes (*n* = 44)	182		

Overall survival time				
Parameter (number of dogs)		PFS (days)	*p*	HR (95% CI)
LGL morphology	No (*n* = 64)	242	0.034	2.87 (1.08–7.62)
	Yes (*n* = 6)	99		
T cell CD4+/CD8+ phenotype	No (*n* = 3)	434	0.059	3.86 (0.95–15.68)
	Yes (*n* = 13)	69		
Neutropenia at diagnosis	No (*n* = 63)	223	0.002	7.74 (2.19–27.37)
	Yes (*n* = 3)	50		
Circulating atypical cells	No (*n* = 53)	222	0.045	3.46 (1.03–11.63)
	Yes (*n* = 3)	69		
Chemotherapy‐induced neutropenia	No (*n* = 24)	154	0.019	0.49 (0.27–8.90)
	Yes (*n* = 44)	259		

Factors negatively impacting OST on multivariable analysis included neutropenia at diagnosis (*p* = 0.004; HR 41.34 [95% CI 3.4–503.4]), LGL morphology (*p* = 0.023; HR 16.34 [95% CI 1.46–183.0]), and CD4+/CD8+ phenotype (*p* = 0.004; HR 18.4 [95% CI 2.55–132.64]). Chemotherapy‐induced neutropenia was associated with improved survival (*p* = 0.042; HR 0.12 [95% CI 0.04–0.94]).

**FIGURE 2:**
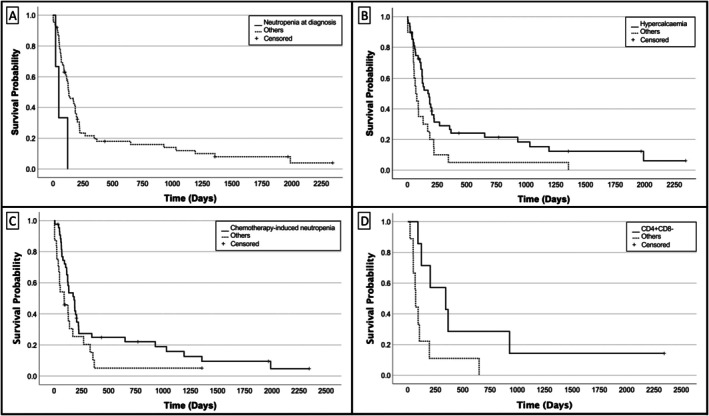
Kaplan–Meier curves for factors significantly associated with progression‐free survival on multivariable analysis in dogs with mediastinal lymphoma treated with multi‐agent chemotherapy: (A) neutropenia at diagnosis (*p* = 0.015), (B) hypercalcaemia (*p* = 0.041), (C) chemotherapy‐induced neutropenia (*p* = 0.014), and (D) CD4+/CD8− immunophenotype vs. others (*p* = 0.004).

**FIGURE 3:**
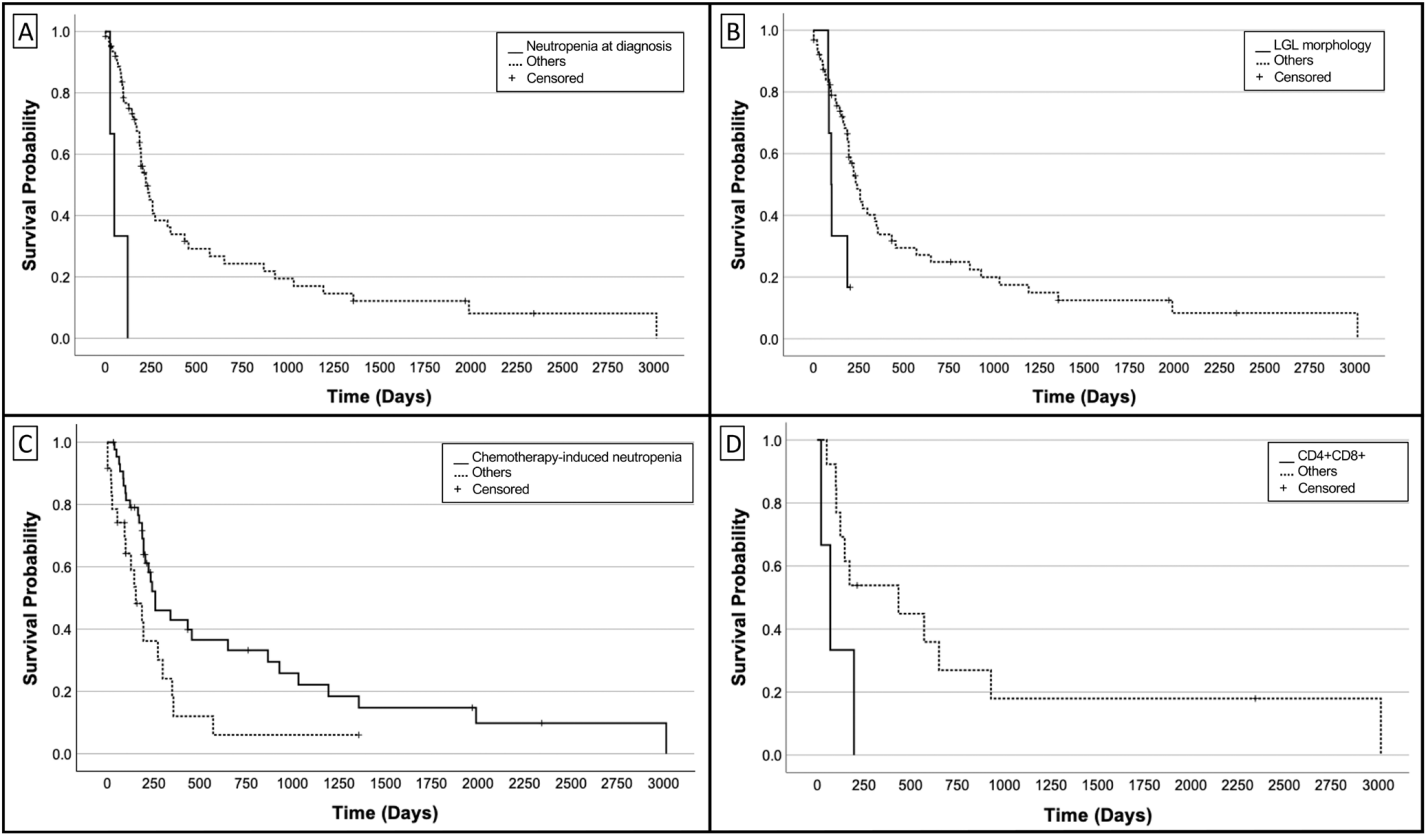
Kaplan–Meier curves for factors significantly associated with overall survival time on multivariable analysis in dogs with mediastinal lymphoma treated with multi‐agent chemotherapy: (A) neutropenia at diagnosis (*p* = 0.004), (B) LGL morphology (*p* = 0.023), (C) chemotherapy‐induced neutropenia (*p* = 0.042), and (D) CD4+/CD8+ immunophenotype vs. others (*p* = 0.004).

Kaplan–Meier survival curves for all variables significant on multivariable analysis are represented in Figures 2 and 3. No variables retained significance on multivariable analysis in relation to clinical response, attainment of complete response, or long‐term survival.

## 4 Discussion

This study describes the largest cohort of dogs with primary mediastinal lymphoma to date. As with previous reports, cases were almost exclusively of T‐cell origin. Whilst no significant differences in outcome were observed between protocols, several prognostic factors were identified, including specific CD4/CD8 expression patterns, neutropenia at diagnosis, chemotherapy‐induced neutropenia, LGL morphology, and hypercalcaemia.

Demographic characteristics of this population align with previous reports, including overrepresentation of Labrador retrievers and boxers, male predominance, and a subset of young dogs including 13 (18.6%) dogs 2 years of age or younger, resembling human T‐LBL presentation [8, 11, 30]. Studies focusing on lymphoblastic lymphoma in dogs also commonly report a mediastinal anatomical form or the presence of a mediastinal mass [12, 13]. Categorisation of lymphoblastic lymphoma in humans relies on phenotypical expression of specific markers such as terminal deoxynucleotide transferase, which is not reliable in dogs [8, 12]. In the present study, the proportion of cases with lymphoblastic morphology (22.2%) appears similar to that reported across the literature for high‐grade T‐cell lymphoma in dogs [13, 26, 31], but contrasts with findings reported by Moore et al. (56.3%) whose study included review of samples by a pathologist [7]. The only morphological characteristic associated with outcome on multivariable analysis was LGL morphology, which has previously been shown to carry a poor prognosis in dogs [32].

Interestingly, 75% of cases with grade reported from histopathology were low‐grade despite demonstrating aggressive behaviour, as reported in canine LGL lymphoma [32]. Grade was not associated with outcome, which may reflect true lack of correlation or limitations inherent to needle core biopsy, possibly not representative [33, 34]. In other tumour types such as soft tissue sarcoma, studies have shown that biopsies can underestimate grade in around 30% of cases [35].

Dogs treated with anthracycline‐based protocols had equivalent response rates, PFS and OST to those reported by Moore et al. where dogs were primarily treated with CHOP [7]. Previous studies have shown that LOP or LOPP may have superior or similar efficacy to CHOP for high‐grade T‐cell lymphoma, and were offered as an alternative to CHOP in some cases [14, 15, 16]. No significant differences in response or outcome were observed in the present study but results should be interpreted with caution due to the small sample size in each group and the non‐standardised treatment protocols. It should be noted that, although previous studies have suggested similar efficacies of CHOP and CEOP and of either 19‐ or 25‐week schedules in dogs with predominantly B‐cell multicentric lymphoma, this has not been investigated specifically in mediastinal lymphoma [36, 37].

Regarding prognostic factors, within cases of T‐cell lineage, CD4+/CD8− phenotype was associated with longer PFS compared to double positive or double negative phenotypes, whilst CD4+/CD8+ co‐expression was associated with poorer survival. Over half of the cases with CD4/CD8 expression investigated were either double positive or double negative, contrasting with nodal T‐cell lymphomas, which are mostly of CD4+/CD8− phenotype in dogs [38]. This aligns with what we understand about normal T‐cell maturation in the thymus, where lymphocytes progress from an initial double‐negative phenotype through a double‐positive stage before differentiating into naïve T‐cells expressing either CD4 or CD8 surface markers [22]. The significance of different immunophenotypes in canine T‐cell lymphomas warrants further investigation, ideally evaluating more specific combinations of cell surface protein expressions, although it may be challenging to include meaningful numbers of dogs receiving standardised therapy.

Neutropenia at diagnosis is not a commonly reported prognostic factor in canine lymphoma, although it has been previously associated with a lower likelihood of chemotherapy response in LGL lymphoma [32]. None of the neutropenic cases in the present study were of granular morphology. Neutropenia could reflect bone marrow involvement which may negatively impact outcome [39]. Routine bone marrow aspirates (performed in a single case in the present study) would be required to corroborate this association and its prognostic significance. Additionally, neutropenia may have influenced treatment decisions impacting dose intensity, which could have contributed to worse outcomes in our population [40]. Conversely, chemotherapy‐induced neutropenia is a well‐established positive prognostic factor that likely reflects optimised dose intensity and/or higher biological effectiveness and was associated with both prolonged PFS and OST [37, 41, 42].

Hypercalcaemia was associated with longer PFS in our study, as with a previous study by Blaxill et al. [14] Historically, hypercalcaemia has been a negative prognostic factor in dogs with high‐grade lymphoma and is typically associated with the more aggressive T‐cell immunophenotype [43, 44, 45, 46, 47]. However, for T‐cell lymphomas specifically, hypercalcaemia does not seem linked to poorer prognosis [6, 7, 15, 48]. Possible explanations for hypercalcaemia being a positive prognostic factor include earlier diagnosis from clinical signs (e.g., polyuria/polydipsia) or an association with an unrecognised phenotype of T‐cell lymphoma with a less aggressive biologic behaviour or greater sensitivity to chemotherapy.

This study's limitations are inherent to its multicentre retrospective nature and study design. The inclusion criteria were designed to select cases of primary mediastinal lymphoma and likely excluded more advanced disease stages, which could present as multicentric forms. This potentially selected dogs more fit to tolerate chemotherapy and excluded cases whose disease severity precluded aggressive treatment. Additionally, cases with mild supradiaphragmatic lymphadenopathy were included based on known human lesion distribution [24]. Whilst this may have introduced some selection bias, it likely reflects the real‐world population of dogs diagnosed with mediastinal lymphoma. Staging was variable and only one dog had bone marrow assessment performed. Consequently, non‐mediastinal involvement could have been unrecognised in some cases and the prognostic significance of bone marrow involvement could not be adequately assessed. Furthermore, whilst hepatic or splenic involvement was permitted and inclusion of hepatosplenic lymphomas with a concurrent mediastinal mass cannot be entirely excluded, primary mediastinal disease was clinically suspected in all six cases of confirmed hepatic involvement. Substage assignment may have been affected by variations in how individual clinicians assessed clinical signs, possibly influencing the ability to evaluate substage as a prognostic factor [49]. Immunophenotyping methods were variable and conducted at different laboratories. Slides were not reviewed for morphological categorisation, and information was not available in some cases. Ideally a standardised review by a clinical pathologist would have been performed for consistent cytomorphological classifications of a larger number of cases, potentially revealing prognostic differences between morphological subtypes. Finally, treatment protocols were not standardised and treatment groups were small, limiting statistical power. Equally, the assessment of many potential prognostic variables and multiple measures of outcome likely increased the likelihood of type I error. Future studies with larger sample sizes assessing the most clinically relevant variables identified here would strengthen our findings.

In conclusion, this study describes the largest cohort of dogs with primary mediastinal lymphoma to date, confirming the predominance of T‐cell phenotype with characteristic CD4/CD8 expression patterns of possible prognostic relevance. Results also confirm previously recognised prognostic factors for high‐grade lymphoma and suggest that hypercalcaemia may have different prognostic significance in T‐cell lymphoma. Although prognosis remains guarded to poor, some patients can achieve long‐term remission with either anthracycline‐based or lomustine‐based protocols. Determining the optimal chemotherapy approach will require larger prospective studies with standardised protocols.

## Funding

The authors have nothing to report.

## Ethics Statement

Ethical approval for data collection was obtained from the Royal Veterinary College's Clinical Research Ethical Review Board (URN SR2022‐0172).

## Conflicts of Interest

The authors declare no conflicts of interest.

## Supporting information


**Table S1:** Full univariable analysis of potential prognostic factors in dogs with mediastinal lymphoma.

## Data Availability

The data that support the findings of this study are available from the corresponding author upon reasonable request.
