# Supplementary material for: Mediastinal Lymphoma in 70 Dogs Treated With Lomustine or Anthracycline‐Based Multi‐Agent Chemotherapy
Source: Vet Comp Oncol. 2026 Mar 31;24(2):382–92. doi: 10.1111/vco.70062 (PMC13161738; doi:10.1111/vco.70062)
Supplement: Supplementary file 1 — Table S1: Full univariable analysis of potential prognostic factors in dogs with mediastinal lymphoma. [file VCO-24-382-s001.docx]

| **Variable** | **Clinical response** | | **Complete response** | | **Progression free survival** | | **Overall survival** | | **Long term survival** | |
| --- | --- | --- | --- | --- | --- | --- | --- | --- | --- | --- |
|  | **P-value** | **OR (95% CI)** | **P-value** | **OR (95% CI)** | **P-value** | **HR (95% CI)** | **P-value** | **HR (95% CI)** | **P-value** | **OR (95% CI)** |
| Clinical | | | | | | | | | | |
| Boxer (Boxer/ others) | † | † | † | † | .441 | 1.33 (0.641-2.78) | 0.665 | 1.18 (0.55-2.55) | .676 | 1.32 (0.36-4.93) |
| Labrador (Labrador/ others) | † | † | .843 | 0.86 (0.19-3.94) | .481 | 0.80 (0.43-1.49) | .255 | 0.67 (0.33-1.34) | .676 | 1.32 (0.36-4.93) |
| Pure breed (pure breed/ mixed or cross breed) | .960 | 1.06 (0.11-10.07) | .415 | 1.94 (0.40-9.49) | .762 | 0.90 (0.44-1.83) | .620 | 0.83 (0.39-1.77) | .343 | 2.83 (0.33-24.16) |
| Age | .206 | 0.81 (0.59-1.12) | .521 | 0.92 (0.72-1.19) | .515 | 1.03 (0.94-1.13) | .286 | 1.06 (0.95-1.19) | .204 | 0.87 (0.70-1.08) |
| Weight | **.044** | 1.10 (1.00-1.21) | .985 | 0.99 (0.95-1.05) | .830 | 1.00 (0.98-1.02) | .555 | 0.993 (0.97-1.02) | .185 | 1.03 (0.985-1.08) |
| Substage b | .585 | 1.90 (0.19-19.06) | **.058** | 6.56 (0.94 - 45.99) | .604 | 0.811 (0.37-1.80) | .709 | 0.84 (0.33-2.12) | .692 | 1.56 (0.17-14.12) |
| Histology/ cytology | | | | | | | | | | |
| Histology grade (low/ intermediate/ high) | † | † | † | † | .732 | 1.14 (0.54-2.42) | .721 | 0.88 (0.42-1.81) | † | † |
| Cell size | .291 | 2.13 (0.52-8.70) | 0.939 | 1.05 (0.31-3.50) | .142 | 1.36 (0.90-2.03) | .307 | 1.25 (0.81-1.93) | **.079** | 0.43 (0.16-1.11) |
| LGL morphology | † | † | † | † | .338 | 1.58 (0.62-4.01) | **.034** | 2.87 (1.08-7.62) | † | † |
| Lymphoblastic morphology | † | † | .434 | 2.67 (0.23-31.07) | **.038** | 2.18 (1.05-4.57) | .472 | 1.32 (0.62-2.81) | .103 | 3.96 (0.76-20.67) |
| Phenotypical expression (all) | | | | | | | | | | |
| B Vs T cell | **0.032** | 42 (1.39-1268.86) | **<.001** | 3.36 (†) | .462 | 0.58 (0.14-2.46) | .181 | 0.37 (0.09-1.59) | 0.999 | † |
| MHCII (present/ absent) | † | † | 0.83 | 0.71 (0.04-14.35) | .846 | 0.91 (0.33-2.5) | .308 | 0.58 (0.21-1.65) | .224 | 3.72 (0.45-31.62) |
| CD5 in T cell phenotype (present/ absent) | † | † | .352 | 4.5 (0.20-106.82) | .738 | 0.82 (0.26-2.63) | .996 | 1.00 (0.28-3.73) | .625 | 1.88 (0.15-23.40) |
| CD3 in T cell phenotype (present/ absent) | † | † | .318 | 5.00 (0.21-117.89) | .742 | 1.24 (0.35-4.42) | .818 | 1.16 (0.32-4.21) | .662 | 0.55 (0.04-8.27) |
| CD79a in T cell phenotype (present/ absent) | † | † | † | - | .318 | 0.52 (0.15-1.86) | .411 | 0.55 (0.13-2.28) | 1 | 1.00 (.063-15.99) |
| CD4+/CD8- (Vs other T cell phenotypes) | † | † | .794 | 1.50 (.07-31.58) | **.031** | 0.29 (0.10-0.90) | .189 | 0.47 (0.15-1.45) | .383 | 2.63 (0.30-23.00) |
| CD4+/CD8+ (Vs other T cell phenotypes) | † | † | .213 | 0.11 (.0.004-3.53) | .116 | 2.98 (0.76-11.66) | **.059** | 3.86 (0.95-15.68) | † | † |
| CD4-/CD8- (Vs other T cell phenotypes) | † | † | † | - | .183 | 2.10 (0.71-6.18) | .803 | 1.16 (0.37-3.56) | .889 | 1.17 (0.13-10.22) |
| Paraclinical (other) | | | | | | | | | | |
| Anaemia (present/ absent) | † | † | † | - | .400 | 1.46 (0.61-3.50) | .385 | 1.52 (0.59-3.93) | .804 | 0.75 (0.08-7.03) |
| Thrombocytopenia (present/ absent) | † | † | .672 | 0.58 (.05-7.16) | .470 | 1.46 (0.52-4.10) | .864 | 1.134 (0.03-4.79) | † | † |
| Neutropenia (present/ absent) | † | † | † | - | **.029** | 3.80 (1.15-12.62) | **.002** | 7.74 (2.19 - 27.37) | † | † |
| Neutrophilia (present/ absent) | † | † | .486 | 1.83 (0.33-10.10) | .212 | 0.65 (0.33-1.28) | .282 | 0.66 (0.31-1.41) | .199 | 2.50 (0.62-10.11) |
| Monocytosis (present/ absent) | .585 | 0.59 (0.09-3.87) | .486 | 1.83 (0.33-10.10) | .964 | 1.01 (0.57-1.81) | .726 | 1.12 (0.61-2.06) | .860 | 1.13 (0.30-4.22) |
| Hypercalcaemia (present/ absent) | .514 | 0.48 (0.05-4.38) | .918 | 1.08 (0.24-4.99) | **.016** | 0.51 (0.29-0.88) | .168 | 0.6 (0.37-1.19) | .465 | 1.69 (0.42-6.83) |
| Increase in creatinine | .954 | 1.07 (0.10-11.11) | .223 | 3.91 (0.44-35.15) | .694 | 1.14 (0.60-2.13) | .619 | 1.19 (0.60-2.35) | .800 | 0.83 (0.20-3.50) |
| Hypoalbuminemia | † | † | .386 | 0.28 (0.02-4.95) | .927 | 0.93 (0.22-3.83) | .749 | 0.79 (0.19-3.30) | .339 | 4.00 (0.23-68.67) |
| Hyperglobulinemia | † | † | † | - | .911 | 1.08 (0.26-4.49) | .891 | .91 (0.22-3.76) | .339 | 4.00 (0.23-68.67) |
| Pleural effusion | .170 | 4.69 (0.52-42.48) | .386 | 1.85 (0.46-7.38) | .516 | 0.866 (0.52-1.45) | .120 | 0.64 (0.36-1.13) | .761 | 1.20 (0.37-3.88) |
| Circulating atypical cells | † | † | † | - | .142 | 2.45 (0.74-8.14) | **.045** | 3.46 (1.03-11.63) | † | † |
| Chemotherapy-induced neutropenia | .404 | 2.05 (0.38-11.08) | .352 | 2.00 (0.47-8.60) | **.050** | 0.58 (0.34-1.00) | **.019** | 0.49 (0.27-8.90) | **.081** | 4.13 (0.84-20.28) |
| Resolution in creatinine elevation | † | † | † | - | .254 | 2.50 (0.52-12.13) | .640 | 1.45 (0.30-6.99) | .265 | 0.17 (0.01-3.89) |
| Treatment | | | | | | | | | | |
| Pre-treatment with steroids | .999 | - | .562 | 1.94 (0.21-18.07) | .634 | 0.85 (0.43-1.68) | .599 | 1.21 (0.60-2.42) | .636 | 1.42 (0.33-6.15) |
| L-asparaginase (yes/ no) | .571 | 0.62 (0.12-3.30) | .185 | 0.39 (0.10-1.57) | .847 | 1.05 (0.63-1.77) | .153 | 0.65 (0.36-1.18) | .713 | 1.25 (0.38-4.10) |
| Protocol (LOP, LOPP, CHOP, CEOP) | .996 | - | .233 | - | .358 | - | .115 | - | .429 | - |
| Rescue treatments (yes/ no) |  | | | | ‡ | ‡ | .192 | 1.51 (0.81-2.80) | .113 | 031 (0.07-1.32) |

**Supplemental table 1:** Full univariable analysis of potential prognostic factors in dogs with mediastinal lymphoma. *‘†’ = could not be calculated due to limited number of events. ‘‡’ = not applicable.*
